# Supplementary material for: Content-rich biological network constructed by mining PubMed abstracts
Source: BMC Bioinformatics. 2004 Oct 8;5:147. doi: 10.1186/1471-2105-5-147 (PMC528731; doi:10.1186/1471-2105-5-147)
Supplement: Additional File 2 — The original results of the above study (non-essential files are deleted to keep the file size under the limit set by BMC bioinformatics). [file 1471-2105-5-147-S2.bz2 › chilibotAdditionalFile2/dip20/505ID10880444E10672/html/ACVR2_BMP2.html]

 


 **ACVR2** and **BMP2** 
  
Found 20 abstracts in PubMed, retrieved 20.  
 

 What does Google say? 
 PDF only 
| .edu only 

---

**Interactive relationship** (e.g. stimulation, inhibition, etc)

**Neutral relationship**- The binding epitopes of BMP 2  [ **BMP2** ]  for BMPR IA type I and BMPR II or ActR II  [ **ACVR2** ]  type II were characterized using BMP 2  [ **BMP2** ]  mutant proteins for analysis of interactions with receptor ectodomains.  Ref: 10880444 EMBO J, 2000
- BMP receptors IA, IB, and II and activin receptor  [ **ACVR2** ]  I were found to be expressed in myocytes, and BMP 2  [ **BMP2** ]  phosphorylated Smad1 and p38 MAPK.  Ref: 11408477 J Biol Chem, 2001
- In this report, we show that the osteoinductive effects of BMP 2  [ **BMP2** ]  in C2C12 cells are mediated by bone morphogenetic protein receptor type IA in combination with both activin receptor type II  [ **ACVR2** ]  and bone morphogenetic protein receptor type II.  Ref: 9417113 J Biol Chem, 1998
- METHODS To identify amino acid side chains involved in receptor binding, a collection of in vitro mutagenized human BMP 2  [ **BMP2** ]  variants was prepared and subjected to interaction analyses with use of the receptor ectodomains of BMPR IA, BMPR II, and ActR II  [ **ACVR2** ]  immobilized on a biosensor system.  Ref: 11263668 J Bone Joint Surg Am, 2001
- Activin receptor  [ **ACVR2** ]  like kinase ALK 1, ALK 2, ALK 3, and ALK 6 were able to reproduce the hypertrophic maturation of chondrocytes induced by BMP 2  [ **BMP2** ] .  Ref: 12082094 J Biol Chem, 2002

**Non-interactive relationship** (e.g. studied together, co-existance, homology, etc.)

- Globin induction by BMP 2  [ **BMP2** ]  and BMP 4 is blocked by co expression of a dominant negative activin receptor  [ **ACVR2** ] , suggesting that either endogenous activin signals are required for BMP mediated induction, or that the truncated activin receptor  [ **ACVR2** ]  interferes with signaling by BMP receptors.  Ref: 7554498 Dev Genet, 1995
- This capacity was positively associated with the expression of fibroblast growth factor receptor 3, bone morphogenetic protein 2  [ **BMP2** ] , and alpha1 II collagen COL2A1, and its loss was marked by the up regulation of activin receptor  [ **ACVR2** ]  like kinase 1 messenger RNA.  Ref: 11465712 Arthritis Rheum, 2001
